# Risk Factors for Mortality among HIV-Infected Patients with Disseminated Histoplasmosis

**DOI:** 10.3390/jof6040326

**Published:** 2020-11-30

**Authors:** Mathieu Nacher, Kinan Drak Alsibai, Audrey Valdes, Romain Blaizot, Philippe Abboud, Magalie Demar, Félix Djossou, Loïc Epelboin, Caroline Misslin, Balthazar Ntab, Antoine Adenis, Pierre Couppié

**Affiliations:** 1 CIC INSERM 1424, Centre Hospitalier AndreeRosemon Cayenne, 97300 Cayenne, France; antoine.adenis@ch-cayenne.fr; 2 DFR Santé, Université de Guyane, 97300 Cayenne, France; romain.blaizot@ch-cayenne.fr (R.B.); pierre.couppie@ch-cayenne.fr (P.C.); 3 Department of Pathology, Centre Hospitalier AndreeRosemon Cayenne, 97300 Cayenne, France; 4 Equipe Opérationnelle d’hygiène hospitalière, Centre Hospitalier AndreeRosemon Cayenne, 97300 Cayenne, France; audrey.valdes@ch-cayenne.fr; 5 Department of Dermatology, Centre Hospitalier Andree Rosemon Cayenne, 97300 Cayenne, France; 6 Service des Maladies Infectieuses et Tropicales, Centre Hospitalier AndreeRosemon Cayenne, 97300 Cayenne, France; philippe.abboud@ch-cayenne.fr (P.A.); felix.djossou@ch-cayenne.fr (F.D.); loic.epelboin@ch-cayenne.fr (L.E.); 7 Laboratory, Centre Hospitalier AndreeRosemon Cayenne, 97300 Cayenne, France; magalie.demar@ch-cayenne.fr; 8 UMR Tropical Biome and Immunopathology, Université de Guyane, 97300 Cayenne, France; 9 Service de Médecine, Centre Hospitalier de l’Ouest Guyanais, 97320 Saint Laurent du Maroni, France; c.misslin-tritsch@ch-ouestguyane.fr; 10 Département d’Information Médicale, Centre Hospitalier de l’Ouest Guyanais, 97320 Saint Laurent du Maroni, France; b.ntab@ch-ouestguyane.fr

**Keywords:** disseminated histoplasmosis, HIV, mortality, risk factors, liposomal amphotericin B, French Guiana

## Abstract

Identifying prognostic factors is important in order to guide the choice of first-line therapy for disseminated histoplasmosis. Our objective was to identify factors associated with death among a cohort of 330 patients compiled over 34 years of clinical practice in French Guiana. Survival analysis was performed with death as the failure event and date of symptom onset as the origin event. Incidence rates were and Cox proportional hazards models were computed. Overall, 330 HIV-infected patients with disseminated histoplasmosis were included in the analysis, with 126 deaths occurring. One-quarter of all patients died within 6 months of the first symptoms. Patients with dyspnea, renal failure, arterial blood pressure < 90 mmHG, and a WHO performance score > 2 had a greater incidence of death. Bivariate analyses showed that patients with increased LDH, low hemoglobin, low serum protein, low CD4 counts, and low platelets tended to have a greater incidence of death. After adjusting for potential confounders, patients with dyspnea, a WHO performance score > 2, serum protein < 60 g/L, and hemoglobin < 8.9 g/dL had an increased risk of dying. The interaction terms showed that patients treated with liposomal amphotericin B had a marked reduction in death among patients with renal failure; among renal failure patients, the elevation of LDH was associated with a significant risk of death.

## 1. Introduction

Since the 1980s, French Guiana, a French overseas territory between Brazil and Suriname, has had a high human immunodeficiency virus (HIV) incidence rate, with overall prevalence in the population exceeding 1% [1]. In this endemic area for *H. capsulatum*, the main acquired immunodeficiency syndrome (AIDS)-defining infection and cause of death is disseminated histoplasmosis [2,3]. Although case fatality has greatly declined over time, disseminated histoplasmosis still represents a common presentation among patients with advanced HIV, a group of patients that remains of particular epidemiological importance [3,4]. Throughout Latin America, histoplasmosis among HIV-infected patients has been estimated to be a major cause of AIDS deaths and a neglected problem due to lack of diagnostic tests. In 2020, the Pan American Health Organization (PAHO) issued the first diagnostic and treatment guidelines for diagnosis and treatment of disseminated histoplasmosis among persons living with HIV [5]. Apart from the lack of rapid affordable diagnostic tests in most of Latin America and the world, the cost of liposomal amphotericin B, a less nephrotoxic formulation of amphotericin B, results in it rarely being used for treatment. Identifying prognostic factors is important in order to guide the choice of first-line therapy between either itraconazole or a formulation of amphotericin B. Few studies have explored these factors in patients with HIV infections. In the present study, our objective was to identify factors associated with death among a cohort of 330 patients compiled over 34 years of clinical practice in French Guiana.

## 2. Methods

### 2.1. Study Design

A retrospective, multicentric study was performed on patients who were confirmed to have disseminated histoplasmosis between 1 January 1981 and 1 October 2014 in the hospitals of Cayenne, Kourou, and Saint Laurent du Maroni. The in-hospital diagnostic facilities are different but the 3 hospitals collaborate (such as for fungal cultures) and share treatment protocols.

### 2.2. Study Population

Co-infections with HIV and histoplasmosis were enrolled in the Histoplasmosis and HIV Database of French Guiana. The inclusion criteria were as follows: confirmed HIV infection; first proven episode of histoplasmosis (European Organization for Research and Treatment of Cancer/Mycoses Study Group (EORTC/MSG) criteria [6]); and age >18 years.

### 2.3. Study Conduct

This database was created in 1992. It includes incident cases of HIV-associated histoplasmosis from the three hospitals in French Guiana. Epidemiological, clinical, paraclinical, immunovirological, and therapeutic data were collected on a standardized case recording form until October 2014. Hospitalized incident cases of HIV-associated histoplasmosis were included. Sex, age, place of birth, symptoms on admission, clinical entrance examination, immunovirological status, standard biological examinations, medical imaging, mycology, pathology, treatment received, and survival data were collected during the study period. Diagnosis of histoplasmosis was performed through direct examination and culture of samples obtained by biopsy or aspiration. Diagnosis was also obtained by histopathology and cytopathology using specific stains (Gomori–Grocott); antigen detection was not available, and molecular diagnosis was not routinely available. Each patient’s general condition was assessed using the WHO performance score which comprises different categories: 0—fully active, no restrictions on activities; 1—unable to perform strenuous activities, but able to carry out light housework and sedentary activities; 2—able to walk and manage self-care but unable to work, out of bed more than 50% of waking hours; 3—confined to bed or a chair more than 50 percent of waking hours, capable of limited self-care; 4—completely disabled, totally confined to a bed or chair, unable to perform any self-care.

### 2.4. Statistical Analysis

STATA© (College Station, TX, USA) was used for statistical analysis. Survival analysis commands were used with failure being the date of death, origin set as the date the symptoms started, and exit time set as the date the patient was last seen. Incidence rates were computed and crude Cox proportional hazards models were computed. The quantitative variables studied were usually categorized based on quartiles in order to balance the class frequencies as much as possible. Missing values were included as one of the categories, and hence they were part of the multivariate model. The proportionality levels of hazards between variable modalities were verified graphically. Multivariate Cox proportional hazard modeling included significant and important variables such as age group and cluster of differentiation 4 lymphocyte (CD4) count, as well as 4 time periods because of the gradual changes in diagnostic capacity and drug availability. The Variance-Covariance Estimator (vce) option was used for clustering based on time period so that the standard errors would allow for intragroup correlation. Interaction terms were tested and the significant interaction terms were retained in the final model with the main effects. Statistical significance was set at *p* < 0.05.

### 2.5. Ethical and Regulatory Aspects

The research was approved by the by the Comité Consultatif pour le Traitement de l’Information pour la Recherche en Santé (CCTIRS) (no. 10.175bis, 6 October 2010), the French National Institute of Health and Medical Research Institutional Review Board (CEEI INSERM) (IRB0000388, FWA00005831 18 May 2010), and the Commission Nationale Informatique et Libertés (CNIL) (no. JZU0061856X, 16 July 2010).

## 3. Results

### 3.1. General Results

**Figure 1:**
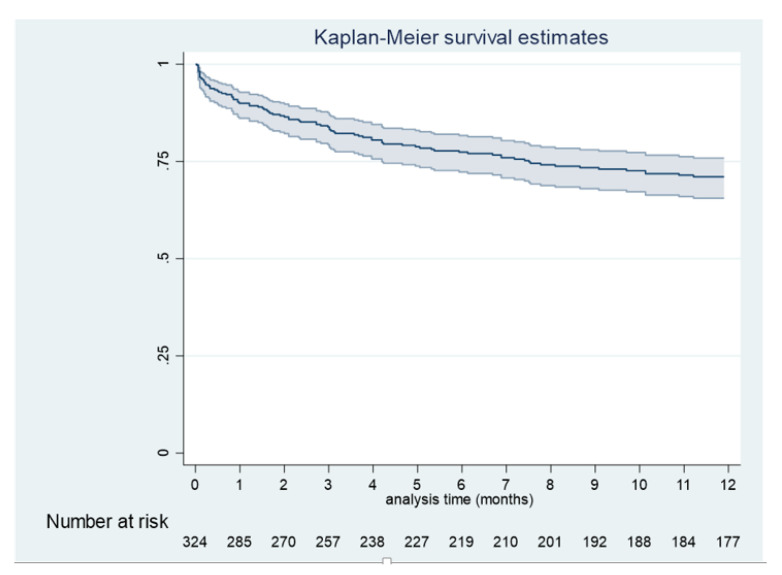
Survival curves for the first 12 months for HIV-associated disseminated histoplasmosis in French Guiana.

Overall, 330 HIV-infected patients with disseminated histoplasmosis were included in the analysis, with 126 failures (deaths) occurring, giving an overall time at risk of 849.9 people/year at risk. The median follow-up time was 1.27 years. One-quarter of all patients died within 6 months of the first symptoms (Figure 1). The mean age was 40 years (±9.7 years). The sex ratio (male/female) was 1.9. Patients had been living in French Guiana for an average of 23.9 years (±16.5 years; 33 years for French citizens (±14.8 years) and 11.5 years for foreign patients (±8.6 years)). The median CD4 count was 31 (interquartile range (IQR): 12–70). Although 132 patients (40%) had a concomitant opportunistic infection, their risk of death was not different from patients without concomitant infections. The concomitant infections observed were: esophageal candidiasis (26), chronic herpes (20), cytomegalovirus infection (19), tuberculosis (18), cerebral toxoplasmosis (18), bacteriemia (15), atypical mycobaterial infection (8), salmonellosis (8), pneumocystosis (7), cryptosporidiosis (3), cryptococcosis (2), bacterial pneumonia (2), and aspergillosis (1).

There was a median interval of 19.5 days (range: 5–105) between symptom onset and hospitalization. Liposomal amphotericin B was initiated within a week after hospital admission in half of the patients and within 3 days in 25% of the patients. Only 40 patients were on antiretroviral therapy at the time of diagnosis, and there was no significant difference in the incidence of death rates at 16.2 vs. 14.6 per 100 people/year in those not receiving antiretrovirals at the time of diagnosis.

### 3.2. Univariate Analysis

Table 1 shows the incidence rates and crude hazard ratios for epidemiological, clinical, and biological variables. Survival improved over the 4 time periods considered. There were no significant differences in survival between males and females and between French citizens and foreign citizens (Table 1). There were very few patients aged over 60 years of age, therefore although the incidence in patients aged >60 seemed higher, the relation was not statistically significant. Patients with dyspnea, renal failure, arterial blood pressure <90 mmHG, and a World Health Organization (WHO) performance score >2 had a greater incidence of death. Crude analyses showed that patients with increased lactate dehydrogenase (LDH), low hemoglobin, low serum protein, low CD4 counts, and low platelets tended to have greater incidence rates of death (Table 1). By contrast, the increase of glutamate–oxaloacetate transferase (GOT) or the decrease of neutrophil counts or prothrombin time were not associated with any apparent differences in the incidence of death (Table 1); while liposomal amphotericin B and itraconazole were associated with a decreased risk of death, deoxycholate amphotericin B (the drug used until 2003 for severe cases of disseminated histoplasmosis) was associated with an increased risk of death.

### 3.3. Multivariate Analysis

Table 2 shows the results for the multivariate model, including interaction terms. After adjusting for potential confounders, dyspnea and a WHO performance score > 2 were associated with an increased risk of death. Patients with serum protein < 60 g/L and patients with hemoglobin < 8.9 g/dL had an increased risk of dying. The interaction terms showed that patients treated with liposomal amphotericin B had a marked reduction in death among patients with renal failure; among renal failure patients, the elevation of LDH was associated with a significant risk of death. Although low hemoglobin as a main effect was a significant risk factor for death, among patients with dyspnea those with hemoglobin concentration < 7.6 had a lower risk of dying than those without.

## 4. Discussion

There have been few studies on prognostic factors, and those that were performed were heterogeneous in design and criteria [7,8,9,10]. Despite this heterogeneity, similar predictive factors were identified here, including respiratory difficulties, alteration of the general condition, and renal failure in patients with elevated LDH. Certain biologic variables have been associated with a poor prognosis, but with poor concordance between studies. As such, low platelets and increased aspartate aminotransferase concentrations were not associated with an increased risk of death in our cohort. CD4 counts were not significantly associated with death, presumably because most patients were profoundly immunocompromised. Low hemoglobin concentrations were associated with increased risk of death, but when studying the interaction term between dyspnea and hemoglobin concentration, there was a surprising reduction of the risk of dying among dyspneic anemic patients, perhaps suggesting that dyspnea in such cases was partly due to anemia and not lung tissue alteration. Patients with low serum protein concentrations (<60 g/L) had an independently higher risk of death than those with higher serum protein concentrations, an observation that is somewhat similar to the observation made by Wheat et al., who observed that decreased serum albumin concentrations were a risk factor for severe histoplasmosis [9].

In 2004, the Center for Disease Control guidelines defined severe cases as temperature > 39.8 °C, systolic blood pressure < 90 mmHg, pO2 < 70 mmHg, weight loss > 5%, Karnofsky performance score < 70, hemoglobin < 10 g/dL, neutrophil count < 1000 cells/mL, platelet count < 100,000 mL, aspartate aminotransferase > 2.5 times normal, bilirubin or creatinine > two times the normal rate, albumin < 3.5 g/dL, coagulopathy, presence of other organ system dysfunction, or confirmed meningitis [11]. There were too many missing pO2 variables in our cohort to include it in the analysis. Fever > 39.8 °C was not associated with a greater risk of death, and systolic blood pressure < 90 mmHg was associated with a greater risk of death in univariate analysis only. In our cohort there were only 9 patients with meningitis, which precluded the use of this variable in the analysis. Coagulation abnormalities (prothrombin time < 70%) were not associated with an increased risk of death, but it is of note that there were many missing values in our dataset.

The multivariate analysis showed that liposomal amphotericin B was associated with an 85% reduction of the risk of death in patients with renal failure. Liposomal amphotericin B was also shown to have superior efficacy in treating severe disseminated histoplasmosis in a randomized controlled trial [12]. It is, however, an expensive drug that is available in French Guiana for this indication, but generally is not available in Latin America, or in most low- and middle-income countries globally [7,13]. The recent PAHO/WHO guidelines for the diagnosis and treatment of disseminated histoplasmosis in HIV-infected patients recommended its use, which will hopefully promote efforts to obtain this drug at lower prices and make it available for all endemic countries [5].

The study limitations were that the data collection covered a long period of time, during which changes in diagnostic facilities, therapeutic arsenal, and awareness about the disease could have led to biases. To mitigate this, we used a variable that captured different time periods and used clustered standard errors. Another limitation was the amounts of missing data for some variables, which predominantly corresponded to the oldest files before from 1998. The analyzes adjusted for this by considering this period as a variable and missing values as a modality. Finally, given the profound level of immune suppression of patients with disseminated histoplasmosis, one could argue that such patients died from other causes than disseminated histoplasmosis. However, the risk of death did not significantly differ between those with or without a concomitant opportunistic infection. Despite the above limitations, to our knowledge this is the largest cohort of patients with disseminated histoplasmosis studied for the risk of death.

In conclusion, despite some limitations, the present cohort is the largest cohort of HIV-associated disseminated histoplasmosis patients used to study the risk of death. Dyspnea, alteration of the general condition, renal failure in patients with elevated LDH concentrations, low hemoglobin concentrations, and low serum protein concentrations were independently associated with increased mortality among patients with disseminated histoplasmosis. Patients treated with liposomal amphotericin B and renal failure had a lower risk of death. These features are largely consistent with previous studies and should help guide physicians to choose between itraconazole and liposomal amphotericin B when patients are admitted in the hospital.

## Figures and Tables

**Table 1:** Incidence rates of death and crude hazard ratios among Human Immunodeficiency Virus-infected patients with confirmed disseminated histoplasmosis.

Variable	Time at Risk (People/Year)	Number of Subjects	Incidence Rate per 100 People/Year	Crude Hazard Ratio (IC95%)
Time period				
<1998	135.1	42	22.9	1
1998–2003	318.4	99	16.9	0.6 (0.39–0.95)
2004–2009	294.3	103	9.8	0.28 (0.16–0.46)
2010–2014	102.1	86	11.7	0.19 (0.10–0.38)
Age category (years)				
<40	460.2	187	15.6	1
40–60	371.1	133	13.4	0.90 (0.63–1.30)
>60	18.5	10	21.5	1.15 (0.42–3.16)
Sex				
Male	546.8	215	13.8	1
Female	303	115	16.4	1.16 (0.81–1.66)
Nationality				
French	325.4	113	17.2	1
Non French	523.8	215	13.3	0.67 (0.47–0.96)
Dyspnea				
Yes	74.2	47	40.4	3.32 (1.01–5.50)
No	256.1	103	12.1	1
Missing	519.6	180	12.5	1.16 (0.75–1.78)
Systolic blood pressure (mm Hg)				
<90	47.9	29	22.9	2.02 (1.01–4.05)
≥90	304.8	136	9.5	1
Missing	497	165	17.3	2.47 (1.62–3.78)
Maximum temperature (°C)				
≥39.8	127	55	8.6	0.71 (0.35–1.43)
<39.8	248.5	107	11.6	1
Missing	474.3	168	18.1	1.97 (1.29–3.01)
Renal failure (creatinine > 120 µmol/L)				
Yes	86.2	46	28.9	1.81 (1.16–2.82)
No	743.9	276	13.1	1
WHO performance score > 2				
Yes	272.3	149	25.3	2.10 (1.48–2.99)
No	577.1	180	9.8	1
Liposomal amphotericin B				
Yes	172.9	92	11.5	0.54 (0.33–0.88)
No	663.9	228	15.3	1
Deoxycholate amphotericin B				
Yes	133	44	18.7	1.82 (1.16–2.84)
No	701.6	275	13.8	1
Itraconazole				
Yes	673.8	260	13.6	0.61 (0.41–0.93)
No	168.37	63	18.4	1
CD4 count < 50				
Yes	486.5	213	17	1.13 (0.90–1.93)
No	359.2	112	11.1	1
Serum protein (quartiles) g/L				
<60	26.1	13	15.3	1.72 (0.53–5.61)
(60–80)	178.2	89	12.3	1.26 (0.58–2.74)
(80–90)	125.2	49	9.5	1.11 (0.47–2.64)
>90	101.9	37	8.8	1
Missing	418.3	142	18.8	2.83 (1.41–5.65)
Neutrophils (quartiles) per mm3				
<800	135.9	83	16.9	0.92 (0.53–1.60)
(800–1780)	246.5	77	10.9	0.87 (0.52–1.48)
(1781–2820)	238	78	12.6	1
>2820	211.7	77	17.4	1.35 (0.83–2.19)
Missing	17.5	15	51.2	2.73 (1.29–5.77)
Platelets (quartiles) per mm3				
<38,000	118.1	79	19.4	1.00 (0.59–1.72)
(38,000–144,000)	219.2	78	14.1	1.07 (0.65–1.74)
(144,001–240,000)	245.9	79	12.6	0.97 (0.59–1.59)
>240,000	249.1	80	13.2	1
Missing	17.4	14	45.7	2.42 (1.11–5.26)
Hemoglobin (quartiles) g/dL				
<7.6	155.6	79	25.1	2.78 (1.60–4.83)
(7.6–8.9)	240.1	85	13.3	1.77 (1.00–3.13)
(8.9–10.4)	258.2	77	7.3	1
>10.4	178.5	76	15	1.74 (0.96–3.13)
Missing	17.4	13	51	4.87 (2.19–10.80)
LDH (median) (U/L)				
<421	435.9	147	8.2	1
421-max	341.7	142	20.7	2.48 (1.66–3.70)
Missing	72.2	41	26.3	2.63 (1.50–4.59)
GOT (median) (IU)				
<55	422.6	161	13.9	1
55/max	406	153	14.2	1.03 (0.71–1.48)
Missing	21.3	16	0.4	2.3 (1.14–4.66)
Prothrombin time				
<70%	126.8	51	13.3	1.36 (0.74–2.50)
≥70%	247.8	119	11.2	1
Missing	475.1	160	17	2.11 (1.37–3.25)
Concomitant opportunistic infection				
Yes	314.8	132	15.2	1.00 (0.70–1.44)
No	535.1	198	14.6	1

**Table 2:** Adjusted hazard ratios for death among HIV-infected patients with confirmed disseminated histoplasmosis.

Variable	Incidence Rate per 100 Person-Years	Adjusted Hazard Ratio (95% Confidence Interval)	*p*
Time period			
<1998	22.9	1	
1998–2003	16.9	1.09 (0.62–1.92)	0.75
2004–2009	9.8	0.46 (0.23–0.89)	0.02
2010–2014	11.7	0.41 (0.29–0.58)	<0.001
Age category (years)			
<40	15.6	1	
40–60	13.4	0.99 (0.45–2.15)	0.98
>60	21.5	1.70 (0.87–3.31)	0.11
Dyspnea (main effect)			
Yes	40.4	11.74 (1.54–89.10)	0.01
No	12.1	1	
Missing	12.5	6.78 (2.07–22.20)	0.002
Renal failure (creatinine >120 µmol/L) (main effect)			
Yes	28.9	0.51 (0.07–3.57)	0.5
No	13.1	1	
WHO performance score > 2			
Yes	25.3	1.95 (1.25–3.03)	0.003
No	9.8	1	
Liposomal amphotericin B (main effect)			
Yes	11.5	0.62 (0.38–1.01)	0.05
No	15.3	1	
Deoxycholate amphotericin B			
Yes	18.7	0.30 (0.05–1.80)	0.19
No	13.8	1	
Itraconazole			
Yes	13.6	0.63 (0.28–1.40)	0.26
No	18.4	1	
CD4 count < 50			
Yes	17	0.94 (0.62–1.43)	0.78
No	11.1	1	
Serum protein (quartiles) g/L			
<60	15.3	3.57 (1.69–7.54)	0.001
(60–80)	12.3	2.38 (0.84–6.75)	0.1
(80–90)	9.5	2.37 (0.69–8.09)	0.16
>90	8.8	1	1
Missing	18.8	2.92 (0.88–9.64)	0.07
Hemoglobin (quartiles) g/dL (main effect)			
<7.6	25.1	14.58 (5.07–41.95)	<0.001
(7.6–8.9)	13.3	6.21 (1.27–30.32)	0.02
(8.9–10.4)	7.3	1	
>10.4	15	16.30 (1.69–157.30)	0.01
Missing	51	25.45 (5.02–129.01)	<0.001
LDH (median) (U/L) (main effect)			
<421	8.2	1	
421-max	20.7	1.50 (0.61–3.67)	0.36
Missing	26.3	1.83 (0.77–4.35)	0.16
Interaction terms			
Renalfailure#LDH	-	8.75 (1.78–43.02)	0.008
Renal failure#Liposomal amphotericin B	-	0.14 (0.06–0.32)	<0.001
Dyspnea#Hemoglobin in first quartile (<7.6 g/dL)	-	0.15 (0.05–0.49)	0.002
